# Spironolactone Attenuates Bleomycin-Induced Pulmonary Injury Partially via Modulating Mononuclear Phagocyte Phenotype Switching in Circulating and Alveolar Compartments

**DOI:** 10.1371/journal.pone.0081090

**Published:** 2013-11-19

**Authors:** Wen-Jie Ji, Yong-Qiang Ma, Xin Zhou, Yi-Dan Zhang, Rui-Yi Lu, Zhao-Zeng Guo, Hai-Ying Sun, Dao-Chuan Hu, Guo-Hong Yang, Yu-Ming Li, Lu-Qing Wei

**Affiliations:** 1 Department of Respiratory and Critical Care Medicine, Pingjin Hospital, Logistics University of the Chinese People’s Armed Police Forces, Tianjin, China; 2 Tianjin Key Laboratory of Cardiovascular Remodeling and Target Organ Injury, Institute of Cardiovascular Disease and Heart Center, Pingjin Hospital, Logistics University of the Chinese People’s Armed Police Forces, Tianjin, China; French National Centre for Scientific Research, France

## Abstract

**Background:**

Recent experimental studies provide evidence indicating that manipulation of the mononuclear phagocyte phenotype could be a feasible approach to alter the severity and persistence of pulmonary injury and fibrosis. Mineralocorticoid receptor (MR) has been reported as a target to regulate macrophage polarization. The present work was designed to investigate the therapeutic potential of MR antagonism in bleomycin-induced acute lung injury and fibrosis.

**Methodology/Principal Findings:**

We first demonstrated the expression of MR in magnetic bead-purified Ly6G-/CD11b+ circulating monocytes and in alveolar macrophages harvested in bronchoalveolar lavage fluid (BALF) from C57BL/6 mice. Then, a pharmacological intervention study using spironolactone (20mg/kg/day by oral gavage) revealed that MR antagonism led to decreased inflammatory cell infiltration, cytokine production (downregulated monocyte chemoattractant protein-1, transforming growth factor β1, and interleukin-1β at mRNA and protein levels) and collagen deposition (decreased lung total hydroxyproline content and collagen positive area by Masson’ trichrome staining) in bleomycin treated (2.5mg/kg, via oropharyngeal instillation) male C57BL/6 mice. Moreover, serial flow cytometry analysis in blood, BALF and enzymatically digested lung tissue, revealed that spironolactone could partially inhibit bleomycin-induced circulating Ly6C^hi^ monocyte expansion, and reduce alternative activation (F4/80+CD11c+CD206+) of mononuclear phagocyte in alveoli, whereas the phenotype of interstitial macrophage (F4/80+CD11c-) remained unaffected by spironolactone during investigation.

**Conclusions/Significance:**

The present work provides the experimental evidence that spironolactone could attenuate bleomycin-induced acute pulmonary injury and fibrosis, partially via inhibition of MR-mediated circulating monocyte and alveolar macrophage phenotype switching.

## Introduction

Idiopathic pulmonary fibrosis (IPF) is a chronic, progressive, interstitial fibrotic lung disease characterized by chronic lung inflammation, disruption of alveolar structure, interstitial fibroblast proliferation, and excessive extracellular matrix synthesis and deposition [1-3]. Although evidence showed that the persistent inflammatory response is associated with progressive development of IPF, therapies currently used for IPF, namely anti-inflammatory or immunosuppressive drugs, are largely ineffective [4]. Therefore, novel therapies capable of targeting inflammation without compromising body’s immunity can still be a challenge in this area.

Macrophages in lung tissue play an important role in the clearance of pulmonary pathogens and steady-state homeostasis maintenance. Emerging evidence suggests that there is a causal link between lung macrophage mediated inflammation and excessive tissue destruction elicited by variety of exogenous stimuli, i.e., silica and asbestos exposure, virus infection, etc., which will ultimately lead to a failure of inflammation resolution, a key feature that progressively promotes the development of lung fibrosis [5-8]. On the other hand, macrophages are a cell population with high plasticity, and display functional diversity during different stage of inflammatory response [9,10]. The activation state of macrophage can be generally characterized as classical activation (M1 polarization) that is associated with a Th1 immune response, or alternative activation (M2 polarization) that is associated with Th2 immune response [11]. In lung tissue, M1-like macrophages are the first line defense in acute lung injury and are later replaced by M2-like macrophages that contribute to tissue repair and fibrosis. It is generally believed during inflammation, myeloid Ly6C^hi^ monocytes contribute to lung macrophage replenishment [9,12]. The results from recent basic studies indicate that manipulation of macrophage phenotype switch might be a potential target for many macrophage mediated disorders [13-15]. 

Recently, Usher and colleagues demonstrated that macrophages from mice lacking myeloid mineralocorticoid receptor (MR), exhibit a transcription profile that mimic alternatively activated macrophages, and are protected against angiotensin II (AngII) induced cardiac hypertrophy and fibrosis [16]. This work provides evidence indicating that MR in mononuclear phagocytes might be a potential target for therapeutic purpose. Based on current evidence, we speculated that pharmacological inhibition of MR with clinically approved drug, may regulate lung macrophage phenotype switching, as well as their progenitors, bone marrow-derived circulating monocytes, and may confer novel therapeutic potential in a murine model of bleomycin-induced acute pulmonary injury and fibrosis.

## Materials and Methods

### Animals

Eight to ten weeks male C57BL/6 mice, weighing 16-18g, were purchased from Laboratory Animal Center of the Academy of Military Medical Sciences (Beijing, China). Animals received human care in compliance with the Regulations for Management of Experimental Animals (Tianjin Municipal Science and Technology Commission, revised June 2004) which was in accordance with Guide for the Care and Use of Laboratory Animals published by the National Institutes of Health (NIH Pub. no. 85-23, revised 1996). All experimental procedures were performed with the authorization of the Animal Use and Care Committee of the Logistics University of the Chinese People’s Armed Police Forces. 

### MR expression in circulating monocytes and alveolar macrophages

 To validate the mRNA expression of MR in mouse circulating monocytes, circulating monocytes from C57BL/6 mice were purified from peripheral blood using a magnetic bead-based kit (EasySep^TM^ Mouse Monocyte Enrichment Kit, Cat No. 19761, STEMCELL Technologies, Vancouver, BC, Canada). The purity of enriched monocytes was confirmed by flow cytometry (see below). Detailed methods for total RNA isolation, reverse transcription, and real-time PCR analysis are shown below.

To validate the protein expression of MR in circulating monocytes and alveolar macrophages, the purified monocytes and cells from bronchoalveolar lavage fluid (BALF) were seeded on glass slides for immunohistological detection of MR. Briefly, the cells were fixed with methanol, followed by permeabilization with 0.1% Triton X-100. Then, the cells were incubated with the primary anti-mouse mineralocorticoid receptor monoclonal antibody (1:200, ab41912, Abcam, Cambridge, MA, USA) at 4°C overnight. To ensure specificity, isotype control (IgG2a) was prepared. For alveolar macrophages, the cells were further incubated with the primary anti-mouse F4/80 antibody (1:200, ab6640, Abcam) at 37°C for 2 h. After washing with 0.01 M PBS, the cells were incubated with tetramethylrhodamine isothiocyanate (TRITC)-conjugated goat anti-mouse secondary antibody [for alveolar macrophage, fluorescein isothiocyanate (FITC)-conjugated goat anti-rat secondary antibody was also added] in dark. Then, cell nuclei were stained by 4,6-diamidino-2-phenylindole (DAPI, Sigma-Aldrich, St. Louis. MO, USA) with light protection. Images were visualized by a fluorescence microscope (Eclipse 80i, Nikon, Tokyo, Japan). The unstained samples and samples stained with the secondary antibody without incubation with primary antibodies were used as negative controls and showed no signal during analysis. 

### Animal model and experimental design

To induce pulmonary fibrosis, mice were lightly anesthetized by inhalation of ether. Bleomycin A5 (2.5mg/kg body weight in 40μl saline) or saline was administered by oropharyngeal instillation as described previously [17]. Animals were then randomly allocated into four treatment groups: 1) 0.9% normal saline (NS) only; 2) bleomycin (BLM) only; 3) bleomycin plus 0.9% normal saline (BLM+NS); 4) bleomycin plus 20mg/kg of spironolactone (BLM+SP). From the day of the administration (day 0), vehicle (0.9% saline), SP (dissolved in 0.9% saline) were delivered by oral gavage once daily, and continued for 21 days. At 1, 3, 7, 14 or 21 days, animals were sacrificed by exsanguinations under sodium pentobarbital anesthesia (10 mice each time point). Blood, BALF and lung tissues were collected for the following assays. 

### BALF analysis

The BALF was collected through an intratracheal cannula with three sequential 1 mL of 0.9% sterile saline and centrifuged at 300 g for 10 min at 4°C. The cell-free supernatant was stored at -80°C for analysis of cytokines. The cell pellet was resuspended in sterile 0.9% saline for total cell counts, differential cell counts, immunohistochemical staining, and flow cytometry analysis. 

### Histological analysis

The left lung (from which no BALF was harvested) was fixed in 4% paraformaldehyde solution for 24h. After embedding in paraffin, 5 μm sections were prepared and stained with hematoxylin-eosin or Masson’s trichrome, and examined on a light microscope (E600POL, Nikon, Tokyo, Japan). For detection of myofibroblasts, α smooth muscle actin (α-SMA, 1:600, A2547, Sigma-Aldrich, St. Louis. MO, USA) immunofluorescent staining was carried out as previously described [18]. For the evaluation of inflammatory response induced by bleomycin, semi-quantitative scoring criteria by Szapiel and coworkers were used in a blinded fashion [19]. Fibrosis and collagen was determined from 10 non-overlapping fields by using digital quantitative analysis (Image Pro Plus software version 4.5, Media Cybernetics, Silver Spring, MD, USA). The lung fibrosis index was defined as the sum of the total area of collagen in the entire visual field divided by the sum of total connective tissue area in the entire visual field.

### Hydroxyproline assay

The collagen content in the whole left lung was determined by analysis of hydroxyproline as previously described [20]. In brief, lung lobes were homogenized in 1 mL of phosphate buffered saline (PBS, pH=7.4) and then hydrolyzed in 1 mL of 6 N hydrochloric acid for 16 hours at 110°C, and neutralized to pH 7.0 with NaOH. Chloramines T reagent (1 mL of 0.5 mol/L) was then added and the samples were left at room temperature for 20 minutes. Then 20% p-Dimethylaminobenzaldehyde solution (dissolved in 3.15 N perchloric acid) was added to each sample, and the mixture was incubated at 60°C for 15 minutes. Absorbance was measured at 550 nm on a NanoDrop 2000c spectrophotometer (Thermo Scientific, Waltham, MA, USA).

### Flow cytometry analysis

Cells from blood, BALF and lungs were subject flow cytometry analysis on a Cytomics FC500 cytometer (Beckman Coulter, Miami, FL, USA). All antibodies were obtained from Biolegend (San Diego, CA, USA). All data were analyzed with FlowJo software (Treestar, Ashland, OR, USA). 

For validation of the purity of magnetic bead-enriched circulating monocytes, anti-mouse CD11b- phycoerythrin (PE) (clone M1/70) and anti-mouse Ly6G- PerCP-Cy5.5 (clone 1A8) were used.

For analysis of circulating monocyte subsets, ethylenediaminetetraacetic acid (EDTA) anti-coagulated whole blood was stained with anti-mouse CD11b- phycoerythrin (PE) (clone M1/70) and anti-mouse Ly6C-FITC (clone HK1.4), incubated for 30 min at room temperature in the dark. Following red cell lysis, samples were analyzed. 

For immunophenotypic analysis of alveolar macrophages (AM), cells isolated from BALF were first centrifuged (10 min at 400 g at room temperature), and the supernatant was discarded to remove dead cells. For each flow cytometry analysis, the cells were first suspended in 0.4% trypan blue in PBS, and the number of live and dead cells was measured using an automatic cell counter (CounterStar^TM^, Rui Yu Biotechnology Co.,Ltd, Shanghai, China). By this method, the number of live cells in each sample is more than 95%. For subsequent flow cytometry analysis, the cells were incubated with anti-mouse F4/80-PE-Cy5 (clone BM8), anti-mouse CD11c- PE-Cy7 (clone N418) and anti-mouse CD206-PE (clone C068C2). Following incubation, flow cytometry analysis was carried out. For immunophenotypic analysis of interstitial macrophages (IMs), lung single-cell suspensions were prepared from lavaged lung (from which the BALF was harvested) to reduce the contamination of AM. In brief, the lower lobe of right lung were minced and incubated with 0.1 mg/mL collagenase solution (type I, Sigma-Aldrich) at 37°C for 60 min. After filtering through 40 μm nylon mesh, similar procedure to remove dead cells was carried out as did during sample preparation for AM analysis, then the cell suspension was stained anti-mouse F4/80-FITC (clone BM8), anti-mouse CD11c-PE-Cy7 (clone N418) and anti-mouse CD206-PE (clone C068C2). Following incubation, samples were analyzed with flow cytometer. Isotype antibodies (clone RTK2758 for F4/80; clone HTK888 for CD11c; clone RTK2758 for CD206; clone RTK4530 for CD11b; clone RTK4174 for Ly6C; clone RTK2758 for Ly6G) were used to detect nonspecific binding. The gating strategies for analyzing AM and IM were according to previous report [21].

### Real-time quantitative polymerase chain reaction (RT-PCR)

Total RNA from purified blood monocytes and lung tissue was isolated using TRIzol Reagent (Invitrogen, Carlsbad, CA, USA) according to the manufacturer’s instructions. Total RNA (2 μg) was reverse-transcribed into the cDNA using a reverse transcription assay (Promega, Madison, WI, USA) in 25 μL of reaction volume according to the manufacturer’s instructions. Real-time PCR was performed with SYBR Green PCR Master Mix (Roche Diagnostics, Indianapolis, IN, USA) on an ABI Prism 7300 sequence detection system (Applied Biosystems, Foster City, CA, USA) in triplicate and according to a two-step PCR protocol (5 min at 95°C, 40 cycles for 30 s at 95°C, 1 min at 60°C). The primer sequences are shown in Table **1**. Relative expression of real-time PCR products were normalized for expression of the β-actin and expressed as transcript fold change over NS mice using the 2^-△△Ct^ method [22]. 

**Table 1:** Primer sequences used in this study.

**Primers**	**Sequences (5’-3’)**	**PCR products (bp)**
MR	GGCTACCACAGTCTCCCTGA	75
	AGAACGCTCCAAGGTCTGA	
Col I	CATGTTCAGCTTTGTGGACCT	94
	GCAGCTGACTTCAGGGATGT	
Col III	TCCCCTGGAATCTGTGAATC	63
	TGAGTCGAATTGGGGAGAAT	
CCL2/MCP-1	TTAAGGCATCACAGTCCGAG	129
	TGAATGTGAAGTTGACCCGT	
TGF-β1	AAACGGAAGCGCATCGAA	63
	GGGACTGGCGAGCCTTAGTT	
IL-1β	AACGTGTGGGGGATGAATTG	130
	CATACTCATCAAAGCAATGT	
Arg-1	AGGAGAAGGCGTTTGCTTAG	115
	AGGAGAAGGCGTTTGCTTAG	
β-actin	CTAAGGCCAACCGTGAAAAG	104
	ACCAGAGGCATACAGGGACA	

Abbreviations: Arg-1, arginase-1, Col I, collagen type I; Col III, collagen type III; CCL2, chemokine (C-C motif) ligand 2; MCP-1, monocyte chemoattractant protein-1; MR, mineralocorticoid receptor; TGF-β1, transforming growth factor β1; IL-1β, interleukin-1β.

### Enzyme-linked immunosorbent assay

The levels of transforming growth factor β1 (TGF-β1), monocyte chemoattractant protein-1 (MCP-1)/chemokine (C-C motif) ligand 2 (CCL2), interleukin-4 (IL-4), and interleukin-1β (IL-1β) in the BALF were measured by commercially available ELISA kits (R&D Systems, Minneapolis, MN, USA), according to the manufacturer’s instructions. 

### Statistical analysis

All data are presented as the mean ± standard error of mean (SEM). Statistical analysis was performed using GraphPad Prism 5.0 software (GraphPad, San Diego, CA, USA). Statistical comparison of multiple groups was performed by one-way ANOVA with Bonferroni post-hoc test or Kruskal-Wallis test followed by Dunn’s multiple comparisons (inflammation score and fibrosis index). A two-tailed *P* value less than 0.05 was considered statistically significant.

## Results

### MR is expressed in in mouse circulating monocytes and alveolar macrophages

**Figure 1:**
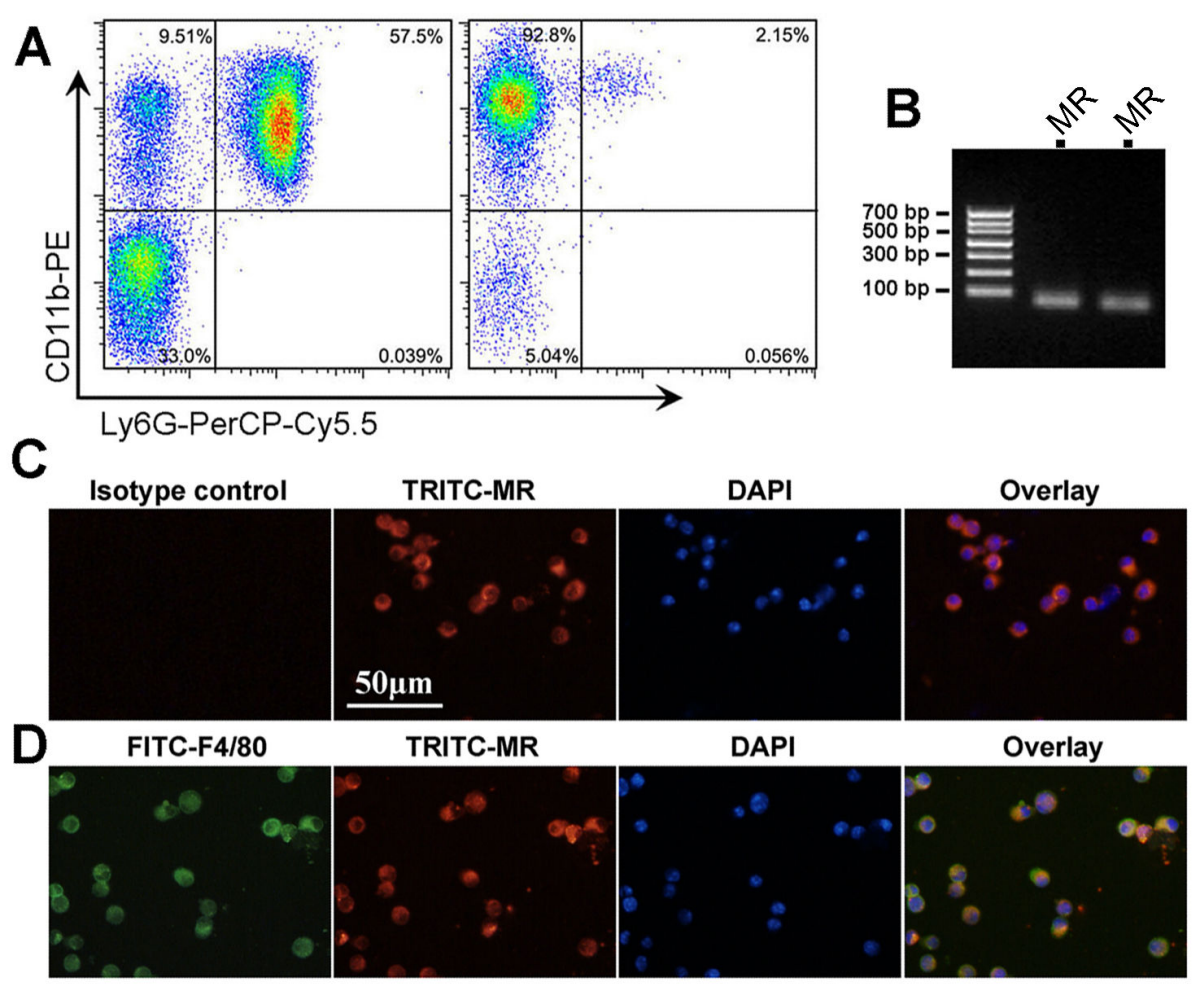
MR is expressed in circulating monocytes and alveolar macrophages. A shows the purity analysis of enriched Ly6G-CD11b+ monocytes by flow cytometry. The left dot plot shows the monocyte (CD11b+ and Ly6G-) purity is 9.51% before enrichment. After enrichment (the right dot plot), the percent of monocytes is 92.8%. B shows the PCR product agarose gel electrophoresis for MR detection after amplification by real-time PCR (from 2 mice, product length 75 bp). Panel C shows the immunofluorescent staining of purified circulating monocytes. Note that all monocytes are positive for MR (red color). Panel D shows the immunofluorescent staining of cells from mouse BALF. Note that cells positive for F4/80 (green) were also positive for MR (red). Abbreviations: DAPI, 4,6-diamidino-2-phenylindole; FITC, fluorescein isothiocyanate; MR, mineralocorticoid receptor; TRITC, etramethylrhodamine isothiocyanate.

By using magnetic bead-based monocyte enrichment method, more than 90% of the harvested cells were Ly6G-CD11b+ (Figure **1A**). Then we confirmed MR mRNA expression in these cells by real-time PCR and PCR product electrophoresis (Figures **1B**). Then, the MR protein expression of enriched monocytes was further validated by immunofluorescent staining (Figure **1C**). Using mouse BALF, we also confirmed MR expression in alveolar F4/80+ macrophages (Figure **1D**). These results suggest that MR is expressed in mouse mononuclear phagocytes, which provides a basis for pharmacological intervention.

### Spironolactone reduces bleomycin-induced alveolitis

**Figure 2:**
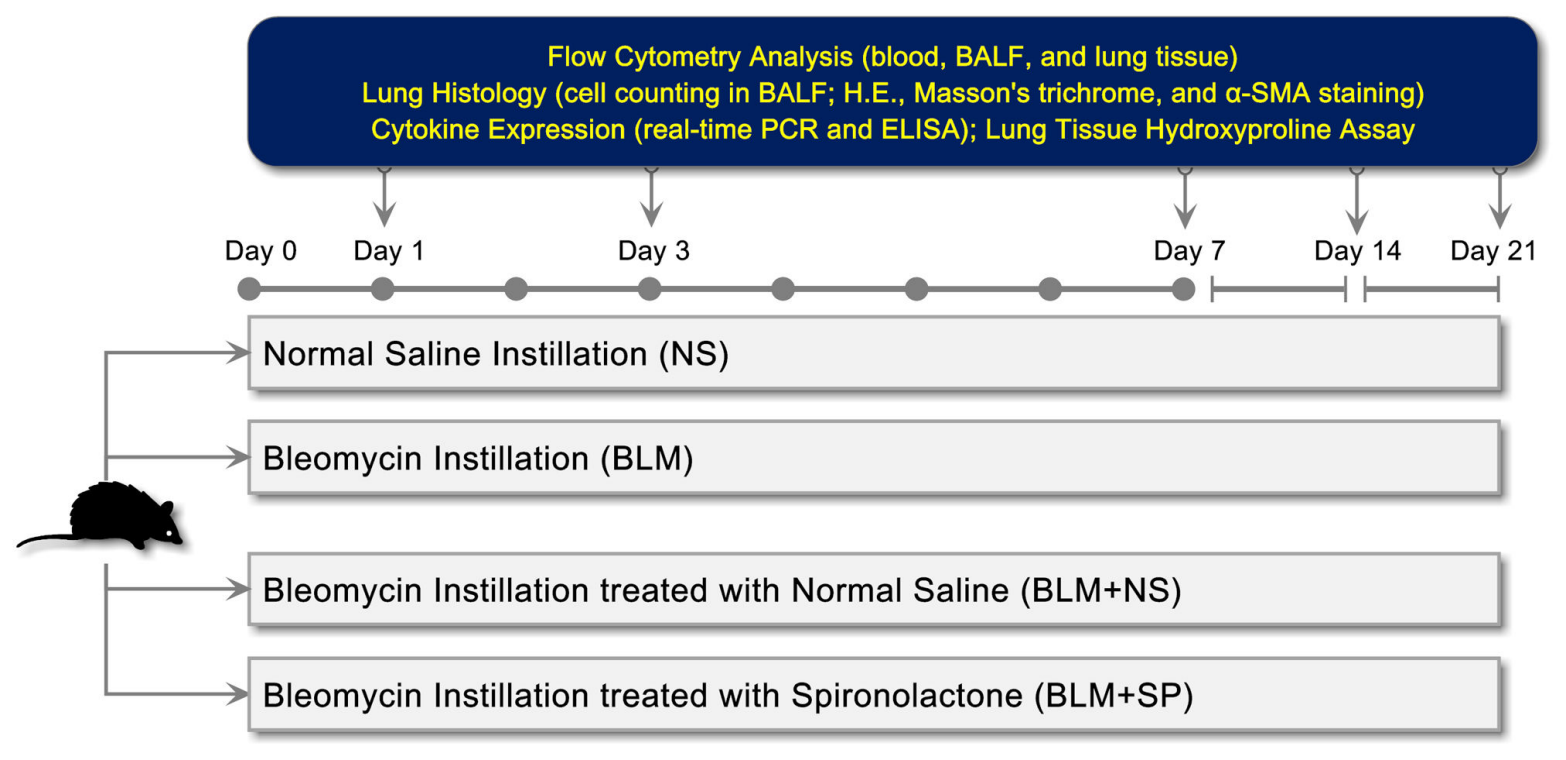
In vivo drug intervention study protocol. Abbreviations: α-SMA, α smooth muscle actin; BALF, bronchoalveolar lavage fluid; BLM, bleomycin; H.E., hemotoxylin and eosin; NS, normal saline; SP, spironolactone.

**Figure 3:**
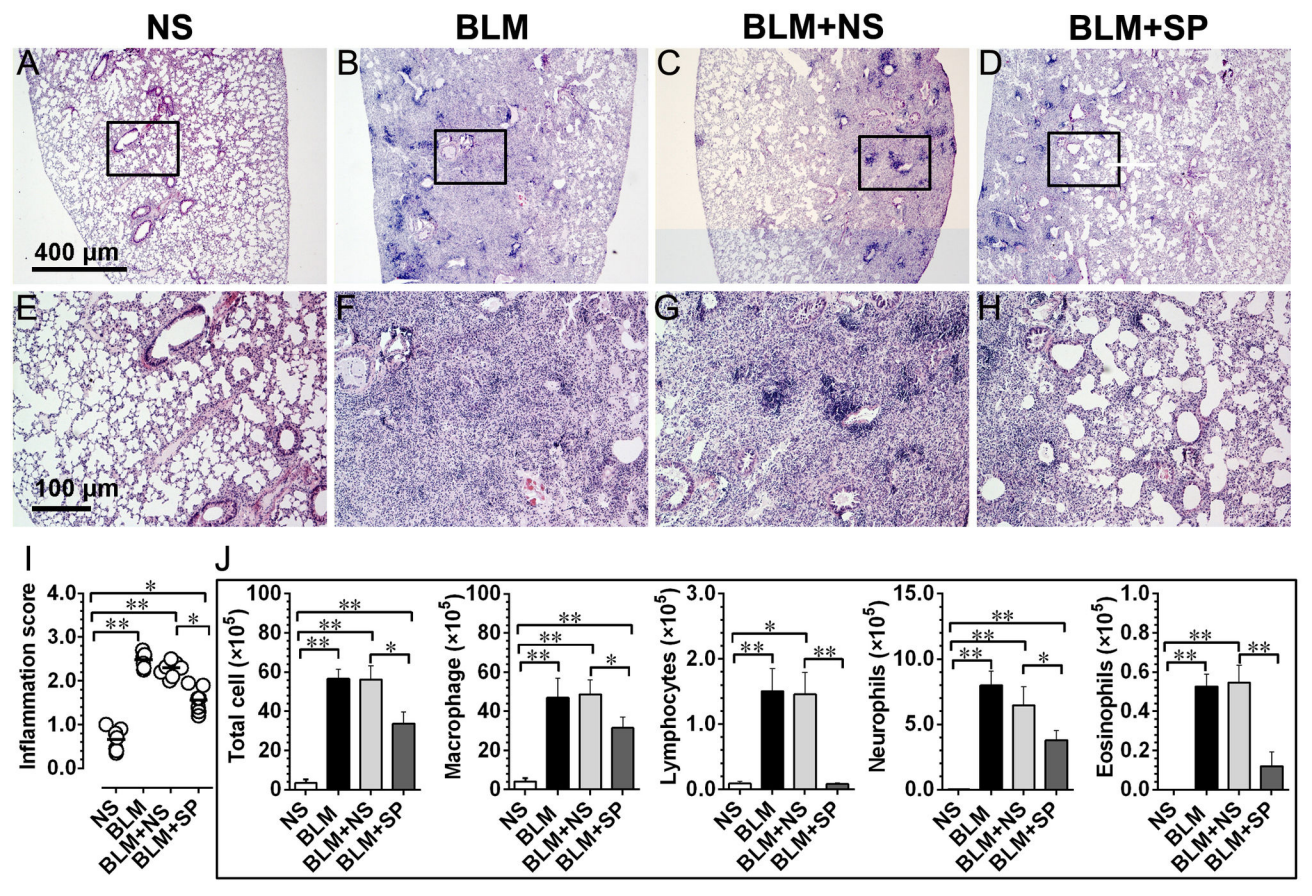
Spironolactone reduces bleomycin-induced alveolitis. Figures A to D, hemotoxylin and eosin (H.E.) stained lung tissue on day 7 after bleomycin challenge. E to H show the magnified fields as indicated by black frames in A to D, respectively. Figure I shows the comparisons of the semi-quantitative inflammation scores across all groups on day 7. Panel J shows the results of differential cell counting from the bronchoalveolar lavage fluid (BALF) that harvested on day 7. Abbreviations: BLM, bleomycin; NS, normal saline; SP, spironolactone. *P<0.05; **P<0.01 (n = 7).

Figure **2** shows the detailed research protocol of in vivo pharmacological intervention study. Figure 3 (A to H) shows the representative H.E. stained lung sections on day 7, which represents the peak magnitude of lung inflammatory response following bleomycin instillation. Spironolactone treatment could significantly reduce the inflammatory response induced by bleomycin (Figure **3I**). 

Panel J in Figure **3** shows the results of differential cell counts from the BALF that harvested on day 7. Typically, the total fluid recovery was over 80% in all animals and the percentages of fluid recovered were not significantly different across all treatment groups. In agreement with histological findings, spironolactone treated lungs exhibited decreased total cell, macrophage, lymphocyte, neutrophil infiltration and esosinophils in alveoli. 

**Figure 4:**
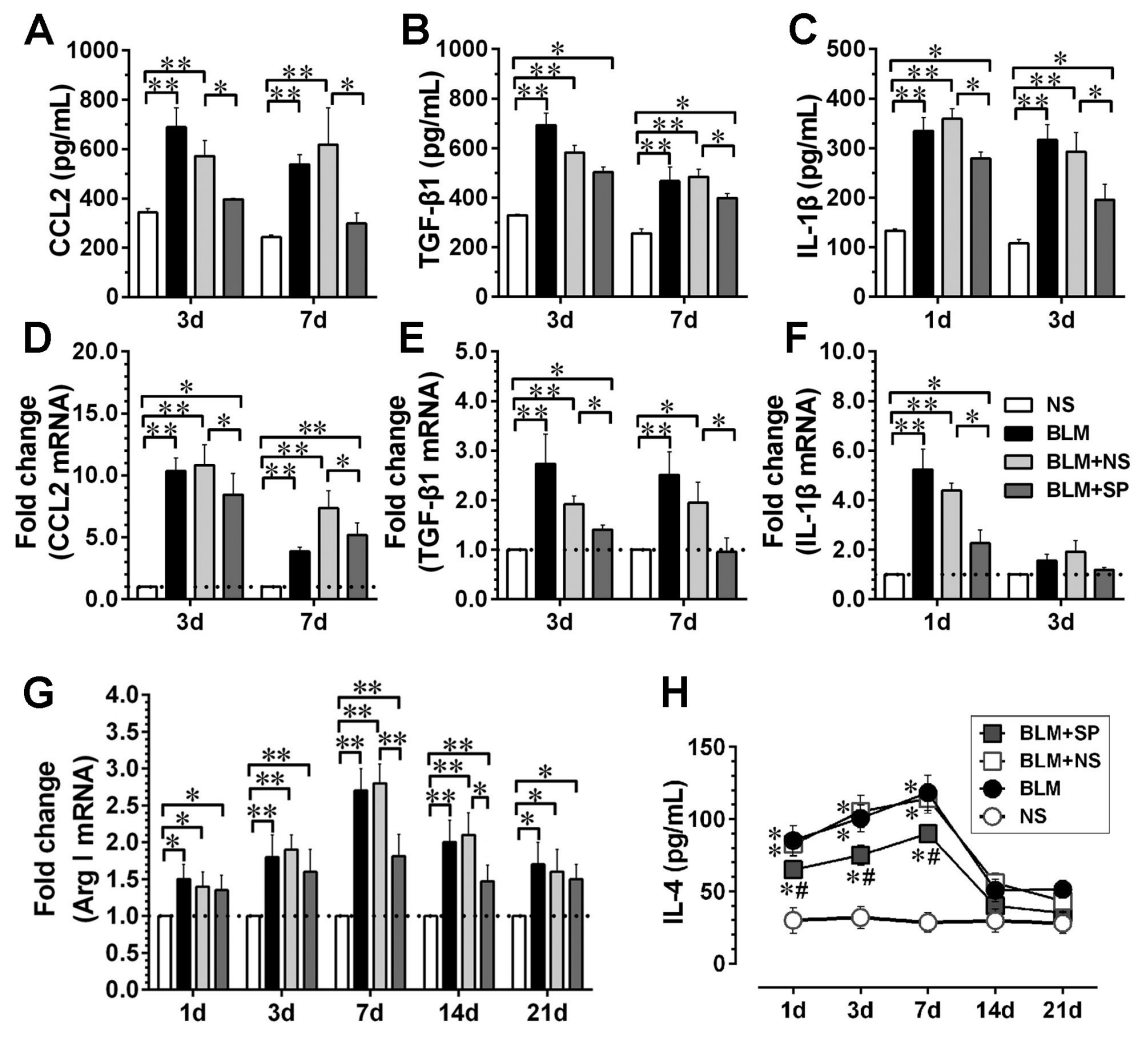
Spironolactone reduces bleomycin-induced inflammatory and profibrotic cytokine expression. Figures A to C show the protein levels of CCL2, TGF-β1 and IL-1βin the bronchoalveolar lavage fluid (BALF) detected by ELISA at selected time points. Figures D to F represent the mRNA levels of above three cytokines in lung tissue at selected time points. G shows the dynamic profiles of Arg-1 mRNA expression in lung tissue; H shows the IL-4 protein dynamics in BALF. Abbreviations: CCL2, chemokine (C-C motif) ligand 2; TGF-β1, transforming growth factor β1; IL-1β, interleukin-1β; Arg1, arginase-1; IL-4, interleukin-4. For Figures A to G, *P<0.05; **P<0.01 (n = 5 to 7); For Figure H, *P<0.05 vs. NS group. #P<0.05 vs. BLM and BLM + NS groups (n = 5 to 7).

Next, we measured the levels of inflammatory and profibrotic cytokines in the BALF and determined related gene expression levels in lung tissue. As shown in Figure **4**, compared with BLM and BLM+NS groups, spironolactone treatment was associated with downregulated CCL2/MCP-1, TGF-β1 and IL-1β both at the mRNA and the protein levels. In addition, markers for M2 polarization, such arginase-1 (Arg-1) mRNA level in lung tissue (Figure **4G**), and IL-4 protein content in BALF (Figure **4F**) were downregulated by spironolactone.

### Spironolactone reduces bleomycin-induced collagen accumulation

**Figure 5:**
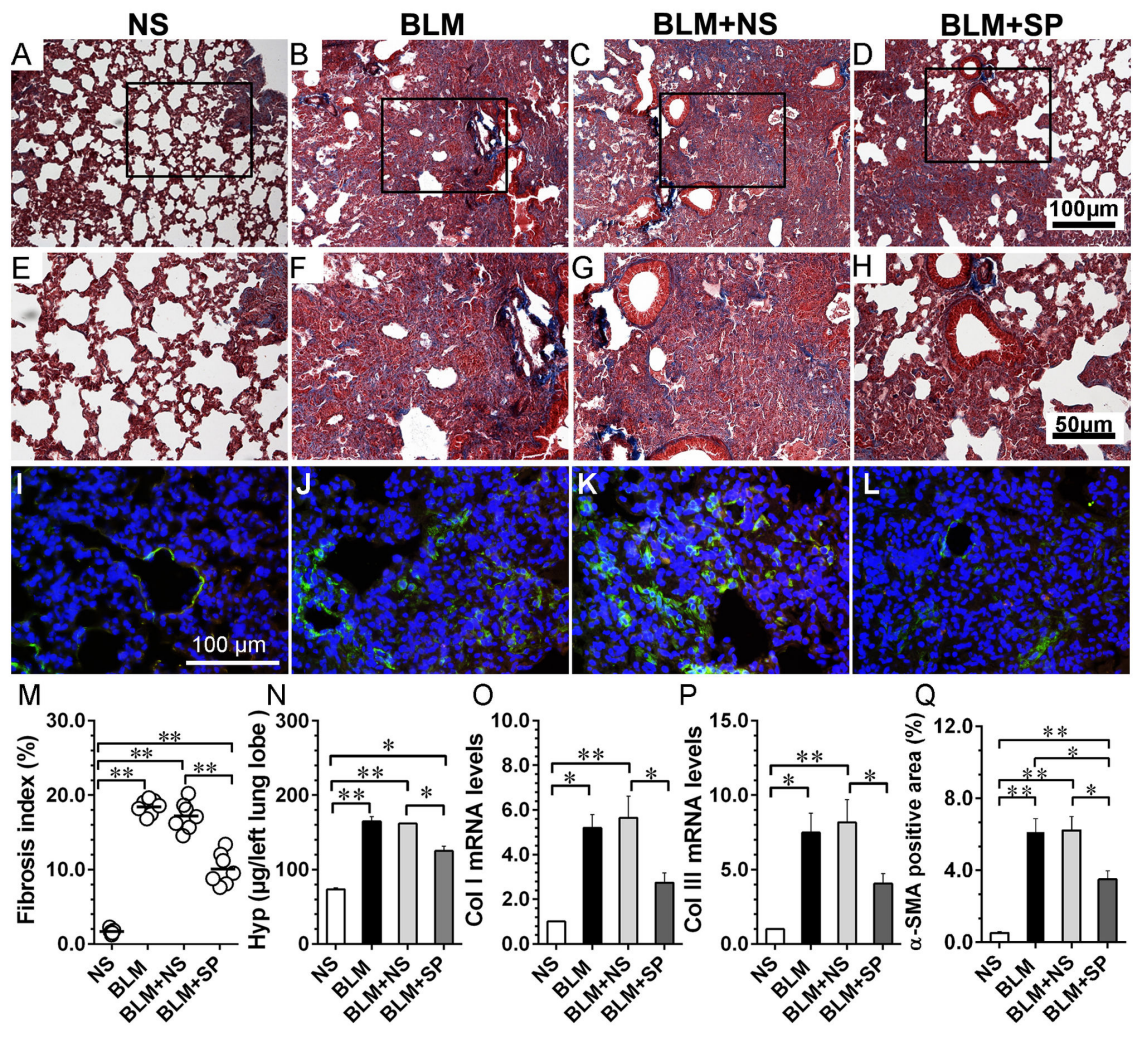
Spironolactone ameliorates bleomycin-induced pulmonary fibrosis. Figures A to D, Masson’ trichrome staining of lung tissue from day 21. E to H show the magnified fields as indicated by black frames in A to D, respectively. Figures I to L show the immunofluorescent staining of α-SMA (FITC-labled, green; nuclei were counterstained by DAPI). Figures M to Q show fibrosis index, total lung hydroxyproline content, collagen type I and collagen type III mRNA expression and α-SMA positive area comparisions in lung tissue from day 21, respectively. Abbreviations: α-SMA, α smooth muscle actin; BLM, bleomycin; Hyp, hydroxyproline; Col I, collagen type I; Col III, collagen type III; NS, normal saline; SP, spironolactone. *P<0.05; **P<0.01 (n = 5 to 7).

Figure **5** shows the profibrotic response using lung tissue that harvested on day 21. The histological analysis showed that MR antagonism was associated with reduced collagen deposition and α-SMA positive cells (myofibroblasts). Compared with NS group, the expression of type I and type III collagen mRNA in the lungs from BLM and BLM+NS groups were significantly upregulated, whereas spironolactone treatment could partially regress bleomycin-induced collagen expression upregulation, which was consistent with the histological findings.

### Spironolactone reduces bleomycin-induced circulating Ly6C^hi^ monocytosis

**Figure 6:**
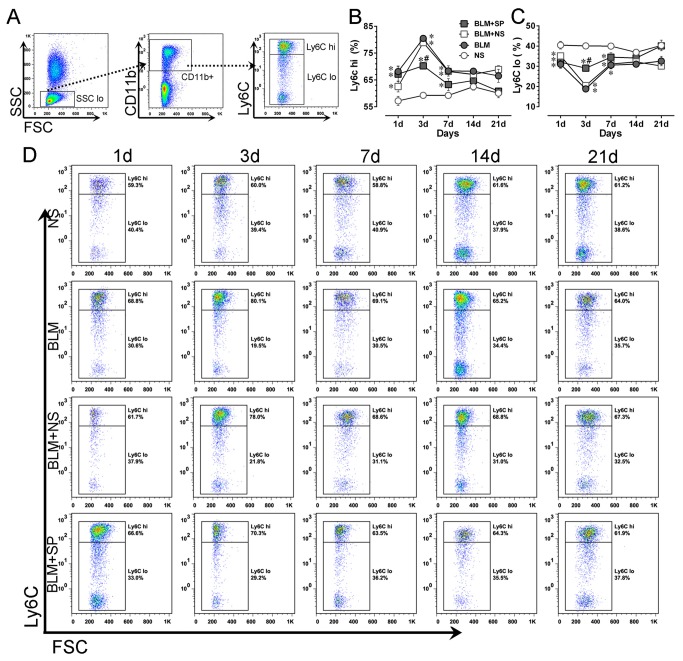
Spironolactone reduces bleomycin-induced circulating Ly6C^hi^ monocytosis. A shows the gating strategies for mouse blood monocyte subsets. B and C show the dynamic profiles of Ly6C^hi^ and Ly6C^lo^ monocytes in all treatment groups, respectively. Panel D shows the representative temporal profiles of flow cytometry analysis (pseudocolor plots) of monocyte subsets in all treatment groups. Abbreviations: BLM, bleomycin; FSC, forward-scattered light; NS, normal saline; SP, spironolactone; SSC, side-scattered light. *P<0.05 vs. NS group. #*P*<0.05 vs. BLM and BLM + SP groups (n = 5 to 7).

We next evaluated the effect of spironolactone treatment on circulating monocyte subset change. Figure **6A** shows the gating strategies for circulating monocyte subset analysis. As shown in Figure **6B**, compared with NS group, BLM treated mice exhibited a significant increase of Ly6C^hi^ monocytes, starting from day 1, reaching the plateau level on day 3, then followed a gradual decrease till day 14. Spironolactone treatment could significantly reduce bleomycin-induced the Ly6C^hi^ monocyte pool expansion on day 3 and thereafter. The reciprocal changes of Ly6C^lo^ monocyte subset is shown in Figure **6C**.

### Spironolactone has no obvious impact on pulmonary interstitial macrophage phenotype

**Figure 7:**
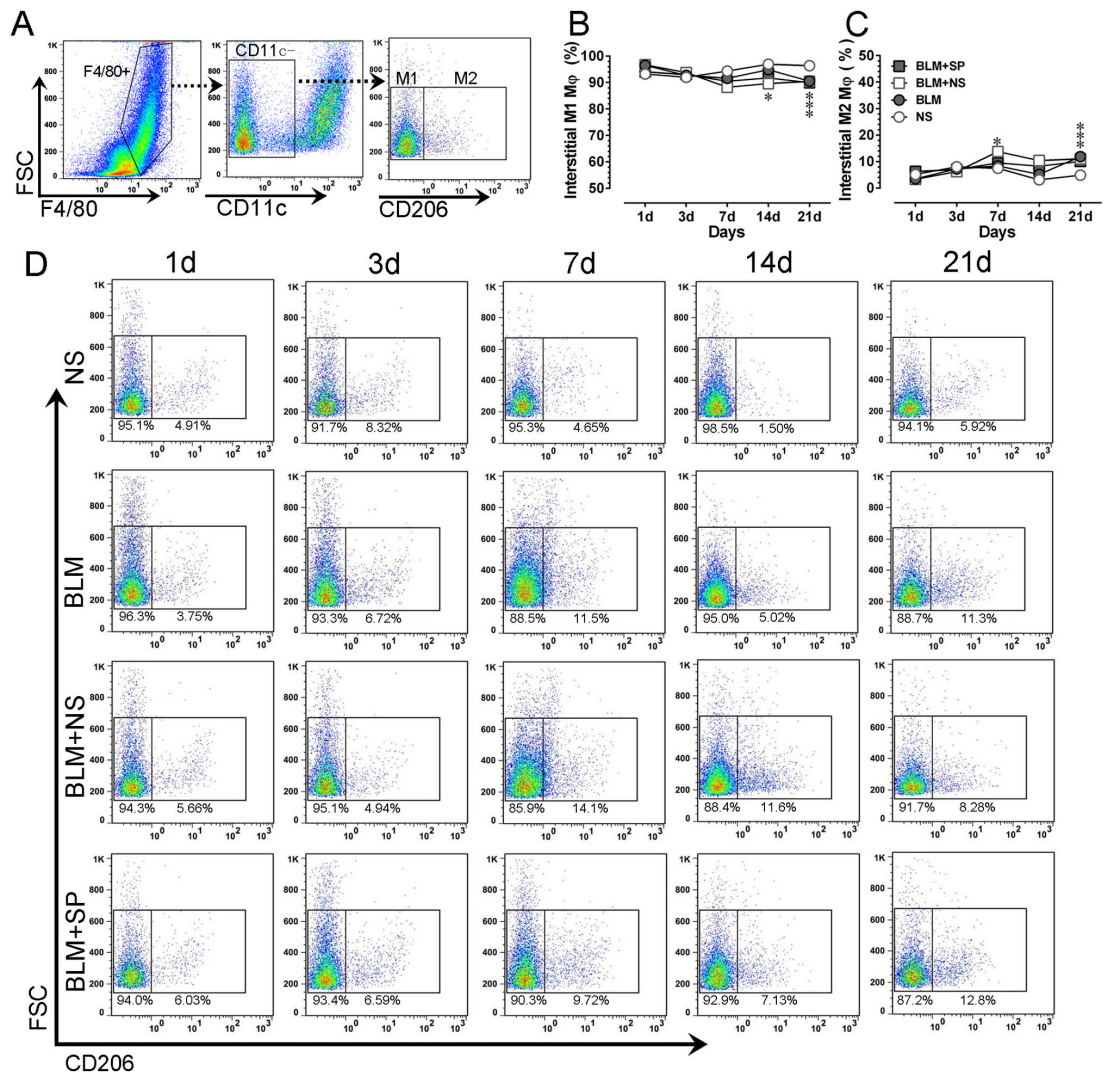
Spironolactone has no obvious impact on pulmonary interstitial macrophage phenotype. A shows the gating strategies for mouse pulmonary interstitial macrophage phenotying after enzymatical digestion. B and C show the dynamic profiles of lung interstitial M1-like (F4/80+CD11c+CD206-) and M2-like (F4/80+CD11c+CD206+) macrophages in all treatment groups, respectively. Panel D shows the representative temporal profiles of flow cytometry analysis (pseudocolor plots) of pulmonary interstitial macrophages in all treatment groups. Abbreviations: BLM, bleomycin; FSC, forward-scattered light; NS, normal saline; SP, spironolactone; SSC, side-scattered light. *P<0.05 vs. NS group. #*P*<0.05 vs. BLM and BLM + SP groups (n = 5 to 7).

Using enzymatically digested lung tissue, we evaluated interstitial macrophage phenotype changes during drug intervention. As shown in Figure **7B**, one day after bleomycin challenge, the majority (more than 90%) of interstitial macrophages presented with a M1-like phenotype (F4/80+CD11c-CD206-), followed by a gradual decreasing trend of the proportion of M1-like macrophages, and this trend reached statistical difference on day 21. Moreover, compared with BLM and BLM+NS groups, spironolactone has no obvious influence on interstitial macrophage phenotype switching induced by bleomycin. 

### Spironolactone partially normalizes bleomycin-induced alveolar macrophage M2 polarization

**Figure 8:**
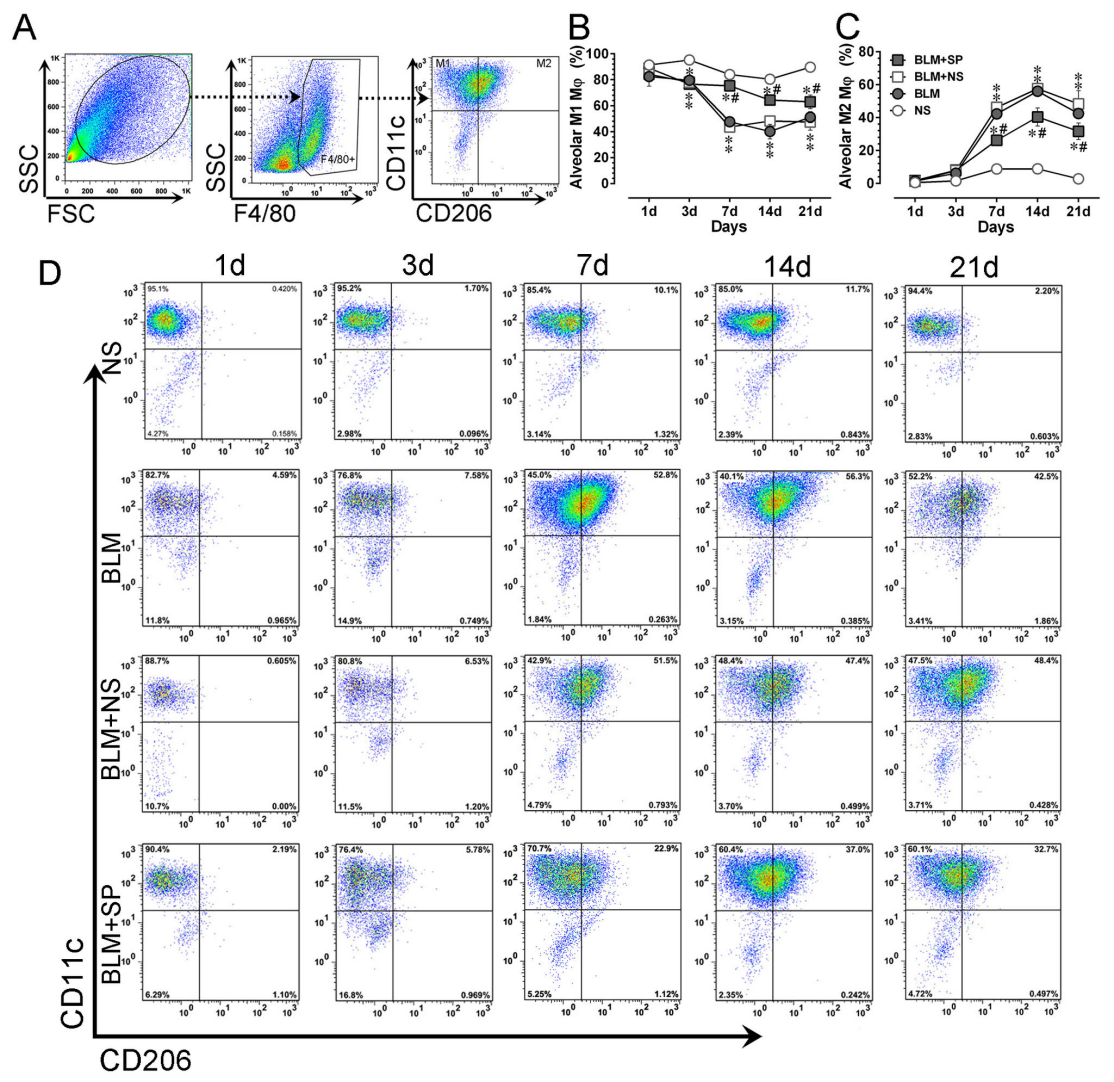
Spironolactone partially normalizes bleomycin-induced alveolar macrophage alterations. A shows the gating strategies for mouse pulmonary alveolar macrophages using lung lavage. B and C show the dynamic profiles of lung alveolar M1-like (F4/80+CD11c+CD206-) and M2-like (F4/80+CD11c+CD206+) macrophages in all treatment groups, respectively. Panel D shows the representative temporal profiles of flow cytometry analysis (pseudocolor plots) of lung alveolar macrophages in all treatment groups. Abbreviations: BLM, bleomycin; FSC, forward-scattered light; NS, normal saline; SP, spironolactone; SSC, side-scattered light. *P<0.05 vs. NS group. #*P*<0.05 vs. BLM and BLM + SP groups (n = 5 to 7).

Then we investigated the impact of spironolactone on alveolar macrophage phenotype changes. As shown in Figure **8B**, alveolar macrophages in NS group were mainly (more than 80%) presented with a M1-like phenotype (F4/80+CD11c+CD206-). After bleomycin challenge, there was a quick decrease of M1-like macrophage with a concomitant increase of M2-like phenotype (F4/80+CD11c+CD206+). Whereas in spironolactone treated mice, this trend was partially normalized, indicating an inhibitory effect on alternative activation by MR antagonism.

## Discussion

Recent studies showed that the renin angiotensin aldosterone system (RAAS) plays an important role in the pathogenesis of lung injury [23-25]. In addition, the therapeutic efficacy of drug intervention targeting this system has been reported in bleomycin-induced lung injury models [26-31]. Zhao and coworker first demonstrated the therapeutical potential of spironolactone in ameliorating bleomycin-induced lung fibrosis [32], which is also supported by a recent study [33]. A growing body of evidence suggests that manipulation of the mononuclear phagocyte phenotype switching could be a feasible approach to alter the severity and persistence of pulmonary injury and fibrosis in experimental models [34-36]. It has been demonstrated that MR plays an important role in regulating myeloid cell phenotype switching in different disease conditions [16,37-40]. To our knowledge, the role of mononuclear cell MR in mediating acute lung injury induced pulmonary fibrosis has not been addressed. The present work confirmed that MR antagonism by a clinically approved drug, spironolactone, could attenuate bleomycin-induced acute lung injury and fibrosis. Specifically, MR inhibition partially attenuates Ly6C^hi^ monocyte expansion in circulating compartment and normalizes disturbed balance of macrophage polarization in alveolar compartment, leading to reduced alveolitis and collagen deposition in lung tissue. These findings highlight mononuclear phagocyte MR as a promising target for ameliorating acute lung injury and profibrotic response in lungs.

The RAAS is a hormone system which acts on multiple physiologic pathways by regulating blood pressure and fluid balance. As the terminal effector of the RAAS cascade, the role of aldosterone/MR signaling has been recently implicated the pathogenesis of cardiovascular diseases, insulin resistance and diabetes, and chronic inflammation associated fibrosis [41-43]. These effects are supported by the fact that in addition to the kidney, there is a wide tissue distribution of MR, such as cardiomyocytes, endothelial cells, vascular smooth muscle cells, adipocytes and macrophages [44]. Here, we demonstrated that MR is expressed both in purified murine circulating Ly6G-/CD11b+ monocytes and in F4/80+ alveolar macrophages, providing a basis for MR regulation of monocyte/macrophage phenotype switching.

Macrophages are professional phagocytic cells with different transcriptional profiles and functional capabilities depending on their origins from various organs [45]. Broadly speaking, the lung tissue contains two tissue-resident macrophage compartments, i.e., alveolar macrophages and interstitial macrophages. The traditional belief that tissue-resident macrophages are derived from circulating monocyte progenitors has been challenged by recent fate mapping studies by showing that the steady-state turnover of alveolar macrophages is extremely low: 8 to 12 months after bone marrow transplantation, 70%-60% of alveolar macrophages are host derived [35,46]. In addition, recent studies demonstrated that lung alveolar macrophages are established prior to birth and maintains themselves subsequently during adulthood independent of replenishment from circulating monocyte input in steady state [47,48].

On the contrary, during acute lung inflammatory response, circulating monocytes have an important impact on the lung macrophage dynamics. In general, recent studies are in agreement with the notion that following injury, there is an increased accumulation of M2-like mononuclear cells in alveoli [10,49-52]. Moreover, in patients with chronic obstructive pulmonary disease, a skewing of alveolar macrophages from an M1 to M2 phenotype has been observed [50,53]. However, with regard to the origin of these M2-like cells, some controversy existed. In an endotoxin-induced lung inflammation model, Maus and coworkers showed that despite a rapid recruitment of monocytes in lung tissue, the resident alveolar macrophage pool remained static throughout the duration of inflammation and the expansion of the lung macrophage pool was mainly mediated by an influx of the circulating monocytes, followed by their differentiation into tissue macrophages [46]. In agreement with this finding, recently Osterholzer et al [54], using a gene-targeted alveolar injury model, demonstrated an increased exudate macrophages and their progenitors, Ly6C^hi^ monocytes, both exhibiting M2 polarization in alveoli. In another study [36], Gibbons and colleagues adoptively transferred Ly6C^hi^ monocytes into bleomycin-treated mice during the progressive phase of lung fibrosis, which led to an exacerbation of disease progression and an increased accumulation of M2-like macrophage in the lung. Surprisingly, these alternatively activated macrophages were host derived and not from the donor Ly6C^hi^ monocytes. As a corollary, regardless of their origins, our current knowledge points to a general scheme of their relationship: initially, acute lung injury induces a rapid expansion and infiltrating Ly6C^hi^ monocytes in lung tissue, which contributes to a paralleled increase of M2-like macrophages (by direct differentiation or by paracrine effects) in alveolar compartment, and the severity and persistency of M2 polarization in alveolar macrophages would ultimately influence inflammation resolution and fibrosis. 

 The above model highlights the circulating Ly6C^hi^ monocytes as a therapeutic target. Although we did not use a monocyte-targeted approach to suppress Ly6C^hi^ monocytosis, it is likely that spironolactone would also exert its major pharmacological effect on circulating monocyte pool since the efficacy of orally administered drug is significantly compromised by its inability to reach alveolar space at an appropriate concentration [55]. Additionally, because evidence shown that monocyte infiltration would facilitates alveolar neutrophil emigration and determines the ongoing neutrophil influx in the persistent phase of acute lung injury [56-58], suppression of Ly6C^hi^ monocytosis by spironolactone would concomitantly lead to a decreased tissue accumulation of neutrophils, which is also observed in our study. 

The present work has the following limitations. First, because spironolactone has anti-androgen effect, the observed effects of this work cannot be totally ascribed to MR antagonism. Indeed, there is a sex discrepancy in bleomycin-induced lung fibrosis, and estrogen may have protective effect on this model [59,60]. In this regard, MR knockout mice are preferred to address this issue. Second, we did not observed significant changes in lung interstitial macrophages by spironolactone. Previous study showed this population might have a role in limiting inflammation and fibrosis [61]. Thus it remains unclear whether CD206 is an appropriate M2 marker for this population as recent study showed that the change of CD206 is modest after bleomycin challenge [51], or this population is insensitive to MR inhibition, or due to enzymatic digestion-induced surface marker loss during sample preparation, a commonly encountered technical issue. Third, due to the wide distribution of MR in the body, the mechanistical explanation of global MR antagonism is fairly complex. For example, aldosterone has been implicated in the pathogenesis of pulmonary hypertension [62], and spironolactone has been shown to attenuate experimental pulmonary hypertension via MR inhibition in pulmonary artery smooth muscle cells [63].Admittedly, bleomycin is also a frequently used tool drug to induce pulmonary hypertension [64,65]. The downregulation of α-SMA by spironolactone observed in this study, also support an anti-fibrotic effect of spironolactone on fibroblasts. Moreover, the functional expression of MR has been demonstrated in neutrophils [66], which may also participate in spironolactone induced amelioration of lung fibrosis, as shown by reduced neutrophil count in BALF. Thus, in addition to its effect on mononuclear phagocytes, the mechanisms underlying therapeutic effect of systemic use of spironolactone on bleomycin-induced lung injury is multifactorial. Forth, it seems obscure to interpret the effect of MR antagonism on alveolar macrophage polarization, since macrophages lacking myeloid MR exhibit alternative activation (M2 polarization), whereas our results showed that MR inhibition could reduce alveolar M2 polarization. It should be noted the long-established binary classification of macrophage in terms of classical (M1) and alternative activation (M2) is based on in vitro studies [67]. Indeed, a recent study demonstrated eplerenone, another clinically approved MR antagonist, promotes alternative activation in human monocyte-derived macrophages [68]. However, macrophages in vivo maintain their plasticity and can alter their phenotype based on the microenvironment, including cytokine milieu among other factors [10]. As pointed early, drug administration via oral route, cannot reach alveolar space at an appropriate concentration. Therefore, the alterations in alveolar macrophage polarization state cannot be ascribed to MR antagonist’s direct effect. It is conceivable that suppression of inflammatory (Ly6C^hi^ subset) monocyte expansion should be the direct effect by spironolactone, which ameliorates lung injury via the “Ly6C^hi^ directed pulmonary alterative activation” mechanism [36]. Thus, future monocyte-targeted approaches, as well as in vitro studies are warranted to elucidate the molecular mechanism underlying suppressed Ly6C^hi^ monocytosis by MR antagonism. Finally, the algorithms used in this study are relatively simple, and may inadvertently contain dendritic cells and eosinophils. More rigorous and sophisticated algorithms have been published and suggested [51,69].

 In conclusion, the present work provides the experimental evidence that MR antagonism by spironolactone could attenuate bleomycin-induced acute pulmonary injury and fibrosis, partially by reducing circulating inflammatory Ly6C^hi^ monocyte expansion and inhibiting alternatively activation of mononuclear phagocyte in alveolar compartment. Our findings highlight MR as a potential therapeutic target to inhibit Ly6C^hi^ monocyte-mediated inflammatory response in acute lung injury and fibrosis.
